# Actions for mitigating the negative effects of patient participation in patient safety: a qualitative study

**DOI:** 10.1186/s12913-024-11154-1

**Published:** 2024-06-03

**Authors:** Michael Van der Voorden, Arie Franx, Kees Ahaus

**Affiliations:** 1 Department of Obstetrics and Gynaecology, Erasmus MC, Doctor Molewaterplein 40, 3015 GD Rotterdam, Netherlands; 2 Department of Health Services Management & Organisation, Erasmus School of Health Policy & Management, Erasmus University Rotterdam, Burgemeester Oudlaan 50, Rotterdam, Netherlands

**Keywords:** Patient participation, Involvement, Engagement, Patient-centered care, Patient safety, Culture change, Obstetrics, Gynaecology, Actions, Negative effects

## Abstract

**Background:**

Recent research within the context of Obstetrics shows the added value of patient participation in in-hospital patient safety. Notwithstanding these benefits, recent research within an Obstetrics department shows that four different negative effects of patient participation in patient safety have emerged. However, the approach to addressing these negative effects within the perspective of patient participation in patient safety is currently lacking. For this reason, the aim of this study is to generate an overview of actions that could be taken to mitigate the negative effects of patient participation in patient safety within an Obstetrics department.

**Methods:**

This study was conducted in the Obstetrics Department of a tertiary academic center. An explorative qualitative interview study included sixteen interviews with professionals (*N* = 8) and patients (*N* = 8). The actions to mitigate the negative effects of patient participation in patient safety, were analyzed and classified using a deductive approach.

**Results:**

Eighteen actions were identified that mitigated the negative effects of patient participation in patient safety within an Obstetrics department. These actions were categorized into five themes: ‘structure’, ‘culture’, ‘education’, ‘emotional’, and ‘physical and technology’. These five categories reflect the current approach to improving patient safety which is primarily viewed from the perspective of professionals rather than of patients.

**Conclusions:**

Most of the identified actions are linked to changing the culture to generate more patient-centered care and change the current reality, which looks predominantly from the perspective of the professionals and too little from that of the patients. Furthermore, none of the suggested actions fit within a sixth anticipated category, namely, ‘politics’. Future research should explore ways to implement a patient-centered care approach based on these actions. By doing so, space, money and time have to be created to elaborate on these actions and integrate them into the organizations’ structure, culture and practices.

**Supplementary Information:**

The online version contains supplementary material available at 10.1186/s12913-024-11154-1.

## Background

Every day, 830 women worldwide die as a result of complications during and following pregnancy and childbirth [1]. Most of these complications are considered preventable and often occur during hospitalization [1–4]. In Obstetrics, this mainly involves severe bleeding and infection after childbirth [1]. Preventable complications occur not only within Obstetrics but also within all specialties and therefore are a reason why patient safety has become an international priority [5–7]. In this regard, patient participation is increasingly used as a strategy to improve patient safety [8–10].

Recent research within the context of Obstetrics indeed shows the added value of patient participation in in-hospital patient safety [11] and more broadly [10, 12, 13]. A common example of patient participation, including Obstetrics patients, is shared decision-making, where the patient is expected to receive sufficient information from the professional and be supported in making medical choices [14, 15]. This can help detect inconsistencies in care [16]. Another example is the use of a surgical safety checklist in cesarean deliveries [17, 18], which can contribute to a reduction in errors and complications [17, 19]. A third illustration is where patients are enabled to monitor their medication and thereby contribute to medication management [20–22], a reduction in medication errors, and improved outcomes [14, 23].

Notwithstanding these benefits, recent research within an Obstetrics department shows that four different negative effects of patient participation in patient safety have emerged [24]. First, involving patients in safety initiatives can lead to anxiety in patients [25]. This includes situations where patients gain a better understanding of medication errors, which increases anxiety [24]. Second, the relationship between the patient and a professional can be negatively affected [26]. Sometimes this occurs because, when the patient and professional do negotiate, there are differences in opinions as to whether the patient’s wishes and needs are medically justified [24]. Third, more responsibility may be placed on the patient than the patient wants [24, 27]. For example, patients may feel they have too much responsibility or that professionals have shifted too much responsibility onto them [24]. Fourth, patient participation in safety initiatives can take up more of the professional’s time [24, 26] since a ‘participating’ patient may pose more questions to healthcare professionals.

To ultimately promote patient safety within an Obstetrics department, it is important to mitigate these negative effects of patient participation in patient safety. To this end, we firstly conducted a general review of the literature on actions that could be taken and classified these according to the model by Bate et al. [28]. This model has six categories of actions: ‘structure’, ‘political’, ‘cultural’, ‘educational’, ’emotional’, and ’physical and technology’ to promote healthcare improvements [28]. The reviewed literature looked at how to deal in general with common problems such as anxious patients [29] or an unsatisfactory patient-doctor relationship [30]. However, the approach to addressing these negative effects within the perspective of patient participation in patient safety is currently lacking. For this reason, the aim of this study is to generate an overview of actions that could be taken to mitigate the negative effects of patient participation in patient safety within an Obstetrics department.

## Methods

### Study design

The aim of this study was to generate an overview of actions that could be taken to mitigate the negative effects of patient participation in patient safety within an Obstetrics department.

To achieve the goal of this research, qualitative research was employed. As a form of qualitative research, an exploratory interview study was conducted to uncover the actions of both patients and professionals within an Obstetrics department. The Standards for Reporting Qualitative Research checklist [31] was used to provide transparency (see Additional file 1).

### Inclusion criteria and participants

This study was conducted within the Obstetrics Department of Erasmus Medical University Center in Rotterdam, the Netherlands. Interviews were held with both patients and birth care professionals to capture their thoughts on appropriate actions to mitigate the negative effects of patient participation on patient safety. Initially, 32 patients and 21 professionals were approached by email, phone, or face-to-face. The inclusion criteria for the patients were that the patient had been admitted to the Obstetrics department, were potentially willing to participate in an interview at least three weeks and no more than six weeks after childbirth, and had mastered the Dutch language sufficiently to fully participate. Inclusion criteria for the professionals were a position as a physician or clinical midwife, at least six months of employment in the Obstetrics department, and sufficient mastery of the Dutch language. A lack of time was the major reason given for nonparticipation by professionals. Patients mostly declined because of insufficient energy after childbirth. We continued to enroll participants until data saturation was achieved. This was achieved once eight patients and eight professionals had been interviewed (see Table 1). Data saturation is reached when the researcher begins to hear the same comments repeatedly within interviews [32]. Within this group of respondents, data saturation was reached because the same actions emerged in the last interviews. This occurred even after the clinical midwife was added alongside the gynecologists.

**Table 1:** Respondents’ characteristics

	Patients *N* (%)	Professionals *N* (%)
Gender		
Male Female	0 (0%) 8 (100%)	6 (75%) 2 (25%)
Age (average)	32 years (range 31–42 years)	51 years (range 37–65 years)
Educational level		
Vocational education Higher vocational education Scientific education	2 (25%) 4 (50%) 2 (25%)	0 (0%) 1 (12.5%) 7 (87.5%)
Profession		
Gynecologist Clinical Midwife		7 (87.5%) 1 (12.5%)

### Data collection

Interviews were conducted between March 2020 and January 2021 by one researcher (MV). Due to COVID-19 concerns, safety measures were observed and the interviews took place on the basis of the patients’ and professionals’ preferences. Nine interviews were conducted face-to-face and seven were conducted by phone. The interviews lasted an average of 59 minutes (range: 43 to 101 minutes) with a focus on forms of individual patient participation. The four negative effects of patient participation on patient safety identified in an earlier study [24] were used as a starting point. The interview topic guide developed for this purpose [24] was also used for this study. In addition, in this study both patients and professionals were specifically asked about actions that could be taken to mitigate these negative effects. The in-depth interviews provided a sense of the local culture in this department. Following the interviews, a member check was carried out by asking the respondents to check for factual inaccuracies in the transcripts. Twelve of the sixteen participants took part in this check. None reported any factual inaccuracies, and no changes were made.

### Data analysis

The texts of the interviews were transcribed, analyzed, and coded by one of the authors using ATLAS.ti V.8 for Windows. ATLAS.ti is a widely used tool to structure qualitative analysis [33] and we opted for deductive analysis because this was an appropriate approach to classify the proposed actions [28]) and generate an accessible overview of the actions identified. The model by Bate et al. [28] was used for this purpose, aiming to systematically identify the actions within the six different categories for healthcare improvement. Because the actions can influence each other and are interdependent, it is suitable to do this according to the classified themes that are interconnected. Firstly, it concerns structural, which involves organizing, planning, and coordinating quality efforts. Secondly, political addresses and deals with the politics of change surrounding any quality improvement effort. Thirdly, cultural entails giving quality a shared, collective meaning, value, and significance within the organization. Fourthly, educational is characterized by creating a learning process that supports improvement. Fifthly, emotional involves engaging and motivating people by linking quality improvement efforts to inner sentiments and deeper commitments and beliefs. Sixthly, it pertains to physical and technological, which involves designing physical infrastructure and technological systems that support and sustain quality efforts [28]. For the coding process, codes were initially assigned to the various actions mentioned by both patients and professionals, enabling us to provide an overview of the actions suggested. Furthermore, this approach provided insight into the level of consensus and the differences and similarities in the actions suggested by patients and by professionals. These actions were then classified according to the six categories proposed by Bate et al. [28]. All the actions suggested by our participants could be fitted within these categories.

## Results

The interviews yielded 18 actions, 13 of which were identified by both patients and professionals. These 18 actions could all be placed in one of five of the six categories proposed by Bate et al. Table 2 below provides a summary of the categories, suggested actions , and whether they were offered by patients, professionals, or both. For an overview of illustrative quotes that most effectively illustrate the story of the results, see Table 3.

**Table 2:** Suggested actions to mitigate the negative effects of patient participation in patient safety

Categories	Actions	Mentioned by
Structure	Appoint a case manager Make time for adequate attention Provide information concerning responsibilities Prepare well for childbirth Clarify role of partner or family	Patients / professionals Patients / professionals Patients / professionals Patients / professionals Patients / professionals
Culture	Patient-centered culture change Encourage patient participation Actively listen to the patient Be transparent Work unambiguously	Patients / professionals Professionals Patients / professionals Patients Patients / Professionals
Educational	Improve negotiation skills Train on shared decision-making Ensure systematic feedback	Professionals Patients / professionals Patients / professionals
Emotional	Share stories Demonstrate leadership Manage expectations	Patients / professionals Professionals Patients /professionals
Physical and technology	Create app for patients’ questions Clarify the patient journey	Patients / professionals Professionals

**Table 3:** Illustrative quotes by both patients and professionals of the suggested actions

Categories	Actions	Illustrative quotes
Structure	Appoint a case manager	*‘’For example, a case manager could be a solution. Not necessarily that you see the same person every time you’re there, but that one person takes a kind of coordinating role over all the appointments. Especially if there are issues, for example with another caregiver, then you can contact them’’* (Patient 7).
	Prepare well for childbirth	*‘’As a patient, you don’t want to get into a discussion about what is going to happen just before delivery. That’s why I’m reminded of that birth plan you draw up in advance. Once childbirth is underway, there is no room to tell or discuss really important things, I think. Anything that is raised with the patient at that time should already be known beforehand. I think through proper preparation you can prevent anxiety’’* (Patient 5).
Culture	Encourage patient participation	‘’*And indeed, when people proactively have their own birth plan, the perception from birth care providers is often that these are ‘difficult’ patients with all kinds of wishes. That really just illustrates that we’re not streamlining it, so from my perspective not discussing it’'* (Professional 6).
	Actively listen to the patient	*‘’The empowerment of today’s patient may already be contributing to that. And then, at the same time, in our culture there has to be a transition to listening to the patient’’* (Professional 5).
	Be transparent	*‘’At some point after delivery I came to the doctor for the follow-up check. At that point, they open my file, in which there is all kinds of information about exactly what happened and whether everything went well. Perhaps nowadays with the new block chain technology they could make sure that there is an online file that you can look into yourself. The goal should be to be able to be more comprehensive and transparent in what is going on with you or what exactly happened. If you have to come up with something anyway, this would be a solution for saving the time of the professionals’’* (Patient 4).
Educational	Train on shared decision-making	*‘’You also see young doctors wanting to do things differently, but you also notice that they do get taught new skills in their training as doctors. These are skills that we used to not get and will have to get if we want to keep up. With us, it is a habit and also a culture that we determine what is good for the patient. The whole transition to the patient as a partner in which we will decide together is going to be a very difficult one. But if we are going to make it, it will also require teaching skills to the patient’’* (Professional 7)
	Ensure systematic feedback	*‘’Perhaps it could be improved by having questionnaires go out periodically, because I personally would find it difficult to address a health care provider about things that are not going well. But I can also imagine that having this information can be very valuable. This can then be done, for example, with grades or sufficient and insufficient. They then have to start correcting on that’’* (Patient 1).
Emotional	Share stories	*‘’I personally think that a sense of safety is still important. So, when you are taken away from your daily situation and admitted [to the birth unit], the feeling of the hospitalized pregnant woman should be one of being well cared for and safe. At that moment, you are anxious, it would be good to test the experiences of patients who have been admitted in a focus group. However, I can imagine that when you are in a room with several people that it can feel unsafe to express your concerns freely’’* (Professional 3).
Physical and technology	Create app for patients’ questions	*‘’That there is sufficient priority and enough capacity, something like that. Or we should think about whether it can’t all be done digitally. That you can send some kinds of questions to a doctor, I can see that too. That you can use an app to send questions to the doctor in advance, so that the doctor can be prepared, and that you can have a kind of structured conversation’’* (Patient 4).

### Structure

The first category ‘structure’ is about establishing working arrangements to prevent negative effects and to ensure patient participation in patient safety should negative effects arise.

#### Appoint a case manager

The respondents mentioned the importance of having a case manager in the primary process as a priority. As soon as patients experience a decrease in trust or the relationship between patient and professional is negatively affected, patients would like to know to whom they can go to discuss the situation. The case manager would then have the task of reassuring patients and ensuring transparency.

#### Make time for adequate attention

Both patients and professionals believed that when a patient’s confidence decreases or the relationship between patient and professional has been affected negatively, it is important that they can engage in a conversation about their anxiety. This requires the professionals to be able to free up time to accomplish this.

#### Provide information concerning responsibilities

To ensure that patients do not feel too much responsibility and that professionals hand over sufficient responsibility, professionals mentioned that it is important to adequately inform patients about the responsibilities of both patients and professionals. When patients know what they are responsible for, they feel more involved in their own care pathway. If errors or deviations in the care pathway are identified by patients, they generally become more anxious and trust may decrease. When this happens, it is important to keep the patient well-informed and provide clarity about the course of action.

#### Prepare well for childbirth

Patients considered this action important so that they can experience as little unnecessary anxiety as possible just before and during childbirth. In doing so, it should be made clear to patients exactly what to expect during childbirth. The interviews highlighted that good preparation for delivery can lead to a better patient experience.

#### Clarify role of partner or family

To maintain a sense of safety for patients in all situations, the professionals said that it is important that they establish protocols and standard information packages to ensure they discuss issues with the partner or contact person of the mother-to-be. Here, it is important that the professional takes responsibility for discussing this, so that the patient does not feel that the onus is on herself to pass on information.

### Culture

The actions within ‘the culture’ category concern ensuring a patient-centered cultural shift, where it is important that professionals work together with the same values.

#### Patient-centered culture change

The suggested cultural changes related to patient-centeredness touch not only on actions within the culture theme, but also within other themes. From the interviews, it was clear that the respondents could conceive actions related to the mindset and motivation of the professionals. Further, what patients find important seems to be receiving minimal attention at present. In addition, patients were given minimal voice in the care process. To mitigate the negative effects, a cultural change is needed through which a patient’s values become the focus of their care.

#### Encourage patient participation

Professionals admitted that they do not always encourage patient participation because they frequently consider patients’ wants and needs as medically irresponsible and of little relevance to the outcome. As a result, professionals may shy away from patient participation. To mitigate the negative effects, it is important that patients are encouraged to participate in a desirable way. The professionals indicated that patients who want to proactively participate can be labeled as difficult.

#### Actively listen to the patient

Here, the professionals indicated that they are not used to actively listening to the patient. Both patients and professionals indicated that active listening is important to hear clearly why patients have anxieties.

#### Be transparent

Patients said that they are very dependent on the information they receive from professionals. Anxiety can be alleviated by openness and transparency. Moreover, patients indicated that it is important to provide full information when there are more questions. Provided this happens, patients indicate that there is less interference from them because they then know enough.

#### Work unambiguously

Unambiguous working was mentioned by both patients and professionals although both have different interpretations of this. From the patients’ point of view, it is mainly about unambiguous policies and not doing things that have not been agreed upon. For professionals, it is more about working with consistent values. That is, as soon as a negative effect arises, it is important that professionals have a consistent way of approaching patients.

### Educational

Actions within the ‘educational’ category are about establishing an educational system that seeks to learn from negative effects in order to make improvements and avoid future negative effects.

#### Improve negotiation skills

The professionals reported that, at the point when patients and professionals start to create a birth plan and the patients and professionals negotiate the patient’s wants and needs and maybe fail to come to an agreement, they require conversational techniques that they do not always possess and therefore need to learn these skills.

#### Train on shared decision-making

Both patients and professionals indicated the need for training to enable them to take a more active role and participate more effectively in patient safety. This training should focus on shared decision-making, aiming to inform both patients and professionals on what responsibility they should take on and what is expected of them.

#### Ensure systematic feedback

Patients and professionals both indicated that healthcare organizations should use a standard questionnaire to continuously examine any negative outcomes and identify improvements that could be made to avoid these. Furthermore, this systematic feedback should be structurally fed back to the professionals in order that they can learn from it.

### Emotional

The ‘emotional’ theme is about sharing experiences and engaging patients by managing their expectations and showing leadership.

#### Share stories

The respondents mentioned that structurally listening to experiences and perceptions is an action that can prevent future negative effects. To establish this process, it is necessary to hold focus groups or open conversations with patients. This should lead to professionals being encouraged to work on making improvements.

#### Demonstrate leadership

Professionals reported that when the relationship between a patient and a professional has been negatively affected, it is important that the professional demonstrates leadership. This requires professionals to continuously explain why something is done, how it is done, and why it makes sense from the professional’s perspective to do it this way. Furthermore, professionals indicated that this requires listening to patients’ objections and that it is the role of professionals to actively address these objections.

#### Manage expectations

Respondents indicated that in situations where confidence decreases, it is important that patients know where they stand and that their confidence is restored. The professionals indicated that they often feel they have to live up to unrealistic expectations, such as in terms of facilities in the birthing room. As a result, patients and professionals may cease to get along. Patients reported here that it is important that boundaries and limitations are indicated in advance.

### Physical and technology

The ‘physical and technology’ category is about ensuring that the negative effects of patient participation in patient safety are actually mitigated.

#### Create app for patients’ questions

Patient participation initiatives related to patient safety result in more questions arising from patients, requiring professionals to spend more time answering them. To make this more efficient, patients suggested developing an app so they could send questions to the professionals in advance. This was with the goal of reducing the time input by professionals. In addition, some professionals indicated that there should be an app that contains all the information that is important for the patient.

#### Clarify the patient journey

Both patients and professionals mentioned that it is important to reduce patients’ sense of bearing considerable responsibility, as this would contribute to managing their expectations during the patient journey. The professional will need to collaborate with an advisor to develop a patient journey that could provide an overview of when and where the patient should obtain appropriate information and therefore know what is expected.

## Discussion

In a previous study, we identified four different negative effects of patient participation in patient safety [24]. To ultimately promote patient safety in an Obstetrics department, this study aims to identify actions to mitigate the negative effects of patient participation in patient safety. These findings are relevant because the approach to addressing these negative effects of patient participation in patient safety within an Obstetrics department is currently lacking. Based on this, Obstetrics departments within hospitals can implement these actions in practice. Within this study, eighteen actions have been identified and four particularly relevant findings are discussed below.

Firstly, the results indicate that the common thread among the eighteen actions is a focus on ‘patient-centered culture change’. Currently, however, this department primarily view it from the perspective of the professionals, rather than adequately considering the viewpoint of the patients. Within this category ‘culture’, various actions emerged: patient-centered culture change, encourage patient participation, actively listen to the patient, be transparent, and work unambiguously. Within this paragraph, further exploration is conducted through comparisons to illustrate the importance of achieving a cultural shift towards the patient’s perspective within this context. An interesting angle here could come from the service dominant logic: that it is not only service providers that create value, but rather that service receivers do so for themselves in use or in collaboration with service providers [34–36]. This involves an evolution where service-dominant logic shifts the focus from goods to services [37]. This consideration, and what can be learned from service dominant logic, has resulted in an application called ‘value-in-use’. Hereby, value is created by the user during the usage of resources, processes (and/or their outcomes) [38]. Translating this to the Obstetrics department of this study, the conclusion could be that participation through patients in safety initiatives within birth care remains at a low level. The respondents indicated that the general line of thought and much of the reasoning is done from the perspective of professionals and does not adequately include the patients’ expertise, knowledge, and thinking. That the patient is not always perceived as a partner is not a surprising outcome, as this has been highlighted in several studies [39, 40]. This is, for example, because patient-centered care in maternity care is perceived differently in practice [41]. Additionally, it is important to acknowledge that effecting such changes within organizations is challenging and requires significant engagement from patients [42] and professionals [43]. Continuing to invest in this area remains valuable, as the literature describes the positive contribution in terms of better outcomes, experiences, and reduced costs [44, 45]. This reflection demonstrates that the underlying theme of this study, aiming for a cultural shift towards patient-centeredness, is valuable.

Secondly, recognizing the importance of achieving a cultural shift towards the patient’s perspective, this section delves deeper into how it is possible to accomplish this within an Obstetrics context.

This involves examining the link with the results of this study, falling under the categories of ‘educational’ and ‘emotional’. Several recent studies have examined how health care organizations can develop patient-centered care and how to implement this in practice [46–49]. A previous study [50] investigated the link between patient safety and patient-centered care within an Obstetrics department, concluding that professionals play an important role in achieving a culture of patient-centered care. In particular, professionals’ knowledge on doing so, demonstration of leadership, academic supervision, mentorship, and financial resources were cited as key components [50]. Looking at this study, demonstrate leadership was indicated by professionals and categorized under ‘emotional’. In practice, professionals often face various challenges in demonstrating leadership [51, 52] Also within the organization where this study took place, efforts are being made to further formalize and strengthen the leadership role, where professionals perform both clinical and management tasks. Various studies indicate that doing so without proper training or preparation is difficult [53, 54], and a structured approach is needed for it to succeed [52]. Additionally, share stories and manage expectations were mentioned in this study. The action of sharing stories could closely relate to systematically gathering feedback and actually taking action based on it in practice. Listening to the stories of obstetric patients aligns well with the idea of driving a culture change towards patient-centered care, by better understanding what they actually want rather than imposing guidelines [55]. At the same time, effectively listening to patients in general is complex and involves various challenges, such as professionals’ time constraints [56]. Moreover, it is noted that receiving feedback and actually acting upon it is also complex [57], thus intersecting with the educational category of actions. Thereby, managing patient expectations is crucial to prepare them for the choices that need to be made [58]. There often appears to be a difference between the expectations of an obstetric patient has for or during childbirth, particularly stemming from the established birth plan, and what actually occurs in practice. This while various professionals observe that unrealistic expectations are included in the birth plan [59]. In this regard, the expectations that patients have can influence patient satisfaction, underscoring the importance of professionals managing patient expectations [60]. This leads to the conclusion that actions in the ‘emotional’ category are complex and require more attention to implement in practice.

Having the right negotiation skills was categorized as an ‘educational’ action in this current study and again was only suggested by the professionals. The desired negotiation skills among professionals are essential for proper interaction with the patient, improving quality, as well as handling tensions or conflicts [61]. Since this is still insufficiently integrated into practice, there needs to be sufficient time and financial investment to make this possible through training(s) [62]. Other actions mentioned within the specific context of Obstetrics in other studies did not emerge as important actions in our study. In this study, two other ‘educational’ actions have been identified: training on shared decision-making and ensuring systematic feedback. Shared decision-making is already being experimented with and integrated within this Obstetrics department. However, both patients and professionals have indicated the need for training to better implement this in practice. The literature also suggests that Obstetric patients do not yet perceive shared decision making as adequately integrated [63]. One reason for this shortfall is the additional time commitment required from professionals on a daily basis [64]. Within this Obstetrics department, a significant amount of patient feedback is already being collected. However, there is currently no effective cycle in place to learn from and improve based on this feedback. Therefore, it can be argued that the feedback is not yet being adequately utilized.

Third, it is notable that within the categories ‘structure’ and ‘physical and technology’, actions emerge that intuitively seem embedded in practice. Under the category ‘structure’, the actions include appoint a case manager, make time for adequate attention, provide information concerning responsibilities, prepare well for childbirth, and clarify the role of partner or family. When it comes to appointing a case manager, this is something that is receiving increasing attention in the practice of the department and the hospital, particularly for patients who, in addition to being pregnant, also have (other) medical diseases. The case manager can be deployed as a point of contact at the individual level to align the care plan with the patient, as well as in collaboration with various other professionals [65]. It can be said that this is still perceived as relatively new within Dutch maternity care [66]. When it comes to making time for adequate attention, providing information concerning responsibilities, preparing well for childbirth, and clarifying the role of partner or family it may seem as if these actions are self-evident and therefore can be applied easily in the practice of an Obstetrics department. Given the often urgent nature of an Obstetrics department, time pressure in such situations can increase. A previous study [67] indicates that when time pressure is higher within an Obstetrics department, professionals feel a stronger need to make decisions themselves. This could explain why both patients and professionals have mentioned all three actions.

Under the category ‘physical and technology’, the actions include creating an app for patients’ questions and clarifying the patient journey. The suggestion of creating an app within this Obstetrics department is somewhat surprising, as such an app for patient questions may already be implemented within the hospital. However, it is possible that its usage is still minimal or that patients and professionals are not sufficiently familiar with it. In a study on the use of eHealth and mobile health within an Obstetric context, it is suggested that it is the role of professionals to involve pregnant women in order to lead to successful integration [68]. Additionally, the results suggest that for managing responsibilities and the expectations associated with them, it is essential to provide better insight into the patient journey. It could be valuable to make the patient journey transparent, with it being the responsibility of professionals to capture the perceptions, preferences, and expectations of the patient upfront [69].

Fourth, our study yielded 18 actions to mitigate the negative effects of patient participation in patient safety within an Obstetrics department in five of these six categories. That is, no one mentioned an action falling within the ‘politics’ category that Bate et al. define as: *‘dealing with conflicts and tensions between different interests and power relations’*. We offer two possible explanations for why politics was not mentioned in our study. First, many respondents within an Obstetrics department were unfamiliar with the topic being addressed in this study and, consequently, may not have been able to put it into a broader perspective and suggest actions in the political sphere. Second, the actions were primarily envisaged from the practical perspectives of the patients and professionals. As such, one could argue that politics as previously defined are largely absent. This can be seen as an interesting result because the literature often discusses tensions that can arise between patients and professionals when there are conflicting interests [70–72]. An example from the obstetric literature suggests that with patient participation in the form of promoting shared decision-making, tension can arise when the patient is challenged to make a choice. However, this may conflict with the clinician’s clinical experience or care standards [73]. Ultimately, this could affect patient safety if the patient prioritizes their own interests over the clinical ones. Another specific example from the obstetric literature shows that among Black American women, a study revealed a sense of powerlessness where doctors played a dominant role in the process [74]. Based on this, it could be argued that there is potentially a ‘politics’ element based on power relations and the interaction between patients and professionals. And it is plausible that in the future, consideration should be given to actions in the ‘politics’ domain, as such tensions may arise in practice.

### Strengths and limitations

First, this study is an inventory off the actions to be taken from the input of both patients and professionals. Because the strength of this is that it allows the conclusion that most of the actions (13/18) were mentioned by both groups. Second, to our knowledge, this is the first study to examine, from the perspective of patient participation in patient safety, the mitigation of negative effects within an Obstetrics department. Thus, it contributes to closing a gap in the scientific literature. Despite these strengths, there are three limitations. Our sample size was limited both in terms of patients and professionals. Additionally, most of the patients were highly educated, and there was no equal distribution among professionals, thus potentially not reflecting the broader population. This might have introduced selection bias [75]. However, additional respondents were recruited until data saturation was achieved. Second, the generalizability of this research is limited, although this is not necessarily a goal in qualitative research [76]. That is, the actions identified come from a specific context and generate an overview of this. Third, by choosing to analyze the data deductively based on Bate et al.’s model [28], the results were shaped by the categories therein. Other models for deductive analysis might have revealed broader or different actions. Nevertheless, the model used does provide specific categories that can then be further elaborated by practitioners.

## Conclusion

Eighteen different actions emerged within five categories from this study in a specific context of an Obstetrics department. No actions fit within the model’s sixth category of ‘politics’. The main finding from this study is that most of the actions highlight the need for a patient-centered culture change. Currently, this still relies heavily on the perspective of professionals and too little consideration is given to that of patients. Future studies could repeat our approach but in a different specific context to see whether other practical actions would be identified for further development. This could include looking at other respondents within the study population, such as other job groups of professionals or less educated patients.

### Practical implications

A specialty or department must recognize that these negative effects occur in patient participation within the realm of patient safety. By doing so, space, money and time have to be created to elaborate on these actions by patients and professionals and integrate them into the organizations’ structure, culture and practices.

### Electronic supplementary material

Below is the link to the electronic supplementary material.

Supplementary Material 1

## Data Availability

All data generated or analyzed during this study are included in this published article.
